# Natural Killer Cell-Derived IL-10 Prevents Liver Damage During Sustained Murine Cytomegalovirus Infection

**DOI:** 10.3389/fimmu.2019.02688

**Published:** 2019-11-15

**Authors:** Alaa Kassim Ali, Amandeep Kaur Komal, Saeedah Musaed Almutairi, Seung-Hwan Lee

**Affiliations:** ^1^Department of Biochemistry, Microbiology, and Immunology, Faculty of Medicine, University of Ottawa, Ottawa, ON, Canada; ^2^Botany and Microbiology Department, College of Sciences, King Saud University, Riyadh, Saudi Arabia; ^3^Center for Infection, Immunity, and Inflammation, University of Ottawa, Ottawa, ON, Canada

**Keywords:** natural killer cells, MCMV, immunoregulation, IL-10, liver damage, inflammation

## Abstract

Natural Killer (NK) cells are lymphocytes of the innate immune response that play a vital role in controlling infections and cancer. Their pro-inflammatory role has been well-established; however, less is known about the regulatory functions of NK cells, in particular, their production of the anti-inflammatory cytokine IL-10. In this study, we investigated the immunoregulatory function of NK cells during MCMV infection and demonstrated that NK cells are major producers of IL-10 during the early stage of infection. To investigate the effect of NK cell-derived IL-10, we have generated NK cell-specific IL-10-deficient mice (*NKp46-Cre-Il10*^*fl*/*fl*^) displaying no signs of age-related spontaneous inflammation, with NK cells that show no detectable IL-10 production upon *in vitro* stimulation. In *NKp46-Cre-Il10*^*fl*/*fl*^ mice, the levels of IL-10 and IFNγ, viral burdens and T cell activation were similar between *NKp46-Cre-Il10*^*fl*/*fl*^ mice and their control littermates, suggesting that NK cell-derived IL-10 is dispensable during acute MCMV infection in immunocompetent hosts. In perforin-deficient mice that show a more sustained infection, NK cells produce more sustained levels of IL-10. By crossing *NKp46-Cre-Il10*^*fl*/*fl*^ mice with perforin-deficient mice, we demonstrated that NK cell-derived IL-10 regulates T cell activation, prevents liver damage, and allows for better disease outcome. Taken together, NK cell-derived IL-10 can be critical in regulating the immune response during early phases of infection and therefore protecting the host from excessive immunopathology.

## Introduction

The immune system has evolved over time to defend the host against pathogens while protecting the host from collateral tissue damage. During infections, various immune cells are activated to exert their allotted roles in order to combat the evading microbes. The recognition of a pathogen by immune cells triggers a cascade of antimicrobial mechanisms that ultimately lead to the clearance of the pathogen (1). NK cells are lymphocytes of the innate immune response that play a critical role in controlling viruses during the early stage of infection (2–5). Subsequently, T cells undergo clonal expansion in an antigen-specific manner upon recognition of a virus-infected cell, which results in the killing of infected cells via their cytotoxic activity. In addition, T cells are capable of providing long-term immunity against the pathogens through memory cell production (6, 7). However, the immune response exerted by either NK cells or T cells during microbial infections must be tightly regulated to maintain sufficient levels of inflammation for pathogen elimination while avoiding excessive tissue injury to the host (8).

IL-10 is a potent immunoregulatory cytokine that ameliorates excessive host-damaging immune responses. IL-10 interferes with the expression of major histocompatibility complex (MHC) and co-stimulatory molecules on antigen-presenting cells (APCs) such as macrophages and dendritic cells (DCs), and also limits the production of pro-inflammatory cytokines (9, 10). Various immune cell subsets, like DCs, macrophages, etc., produce IL-10, but regulatory T cells (Tregs) were known to be one of the major IL-10 producers (9). Notably, there have been studies showing IL-10 production by NK cells during various models of infections such as MCMV, *Toxoplasma gondii, Leishmania donovani*, and HIV (10–16).

IL-10 has paradoxical functions during viral infections. During acute influenza infection, blocking IL-10 response resulted in increased pulmonary inflammation and increased mortality (17). Moreover, IL-10 blockade during acute herpes simplex infection resulted in severe disease and enhanced stromal keratitis (18). Acute respiratory syncytial virus (RSV) and MCMV infections of IL-10-deficient mice are associated with increased weight loss and disease severity (19–21). Altogether, these studies demonstrate that IL-10 plays a critical role in limiting T cell-mediated inflammation and injury. On the contrary, blockade of IL-10 was often known to enhance T cell response, resulting in enhanced protection. IL-10 can interfere with protective immunity following a high-dose influenza challenge by inhibiting Th1 and Th17 responses, and blockade of IL-10 protects naïve mice against an otherwise lethal influenza challenge (22). Furthermore, during persistent LCMV infection, IL-10 deficiency resulted in a robust effector T cell response and the rapid elimination of the virus (23). Taken together, IL-10 can be beneficial to the host by protecting from excessive disease-mediated immunopathology, or it can be detrimental to the host by inducing immunosuppression and thereby allowing pathogen persistence.

Generally, the human cytomegalovirus (HCMV) infection is asymptomatic in healthy individuals but can result in fatal consequences in transplant recipients, immunocompromised individuals, and neonates (24). The murine cytomegalovirus (MCMV) is a herpesvirus that replicates in visceral organs such as the spleen and liver (25, 26). During the early phases of acute MCMV infection, NK cells and monocyte/macrophages are quickly recruited to the infected organs (27, 28), and IFNγ production by NK cell limits MCMV infection (29). During the later phases of MCMV infection, CD8 T cells (30), and CD4 T cells (31, 32) accumulate in organs such as the liver and participate in antiviral responses. Together, these immune responses are protective; however, they can lead to disease-mediated pathology if inadequately regulated (20, 25, 26, 33, 34). Many studies have focused on characterizing the robust pro-inflammatory immune responses during MCMV infection; nonetheless, the signals involved in dampening these immune responses have not been completely elucidated.

Several reports have investigated the immunoregulatory role of IL-10 in the context of MCMV infection (20, 35–37). B cells produce IL-10 to prevent an exaggerated immune response by MCMV-specific CD8 T cells (38). Another study demonstrated that CD4 T cells production of IL-10 leads to increased viral burden, allowing MCMV to persist in mucosal areas such as the salivary gland (35). IL-10 was also shown to limit systemic levels of IFNγ, reduce T cell response, and increase the viral loads in the spleens of MCMV-infected mice (20). Administration of recombinant IL-10 in IL-10-deficient mice diminished the levels of pro-inflammatory cytokines and reduced liver pathology caused by MCMV infection (39). In order to evade recognition by T cells, MCMV induces the downregulation of MHC molecules on macrophages through an IL-10-dependent mechanism (37).

Previously, we proposed that the NK cell-derived IL-10 limits the magnitude of the CD8 T cell response in immunocompromised perforin-deficient (PKO) mice by blocking IL-10 during the peak response of IL-10 production by NK cells (36). Nevertheless, since several cell types are capable of producing IL-10 during infections, it remains unclear whether NK cell-derived IL-10 plays a definitive role in the regulation of inflammatory processes during sustained MCMV infection. In this study, we took advantage of a genetic approach and investigated the NK cell-mediated regulatory pathway during MCMV infection by generating an NK cell-specific IL-10-deficient mouse (*NKp46-Cre-Il10*^*fl*/*fl*^). The MCMV infection resulted in a heightened immune response in the spleens and livers of *NKp46-Cre-Il10*^*fl*/*fl*^ mice, in mice deficient in perforin, but not under immunocompetent condition. While viral clearance was not improved, the augmented immune response led to increased liver damage, suggesting the occurrence of excessive immune-mediated pathology.

## Materials and Methods

### Mice and Genotyping

B6.129S6-*Il10*^*tm*1*Flv*^/J mice (IL-10-GFP; *tiger*) and C57BL/6-*Prf1*^*tm*1*Sdz*^/J (perforin-deficient, PKO) mice were purchased from The Jackson Laboratory, USA. IL-10-GFP mice were generated by insertion of an internal ribosomal entry site (IRES) and green fluorescence protein (GFP) element upstream of the polyadenylation site of the *Il10* gene as previously described (40). *Il10*^*fl*/*fl*^ mice were generated by inserting the loxP sequences between parts of the promoter region, transcription initiation site, and the first exon of *Il10* allele (41). The *NKp46*^*iCre*^ knock-in mice were generated by homologous recombination in which improved Cre (*iCre*) was inserted at the 3' end of the *Nkp46* gene (42). The *Ly49h*-intron primers were *D6Ott151*-forward (F): 5'-GTGCTACCACTGAAAACCATTG-3' and *D6Ott151*-reverse (R): 5'-CTGTCTCTTGAGTCACCTGCAC-3' (43). The *Il10* floxed and *Il10* deleted alleles were genotyped using the following primers: F, 5'-CCAGCATAGAGAGCTTGCATTACA-3'; floxed-R, 5'-TCCTCTTGGGATCCAGTTGT-3'; and deleted-R, 5'-GCTGCTTCTCCTGCTGAGTT-3'. Experiments were performed using littermate mice by mating *NKp46-Cre*^+/−^ × *Il10*^*fl*/*fl*^ mice with *NKp46-Cre*^−/−^ × *Il10*^*fl*/*fl*^ mice. All mice were bred and kept in the specific-pathogen-free animal facility at the University of Ottawa in agreement with guidelines and regulations of the Canadian Council on Animal Care. All procedures were approved by and conducted in accordance with the animal guidelines of the University of Ottawa. Unless indicated otherwise, all mice used for experiments were between the ages of 6–12 weeks old.

### MCMV Infection and Virus Titer Determination

MCMV stocks (Smith strain) were generated in our laboratory from the salivary glands of infected BALB/c mice. To determine the kinetics of IL-10 production by NK cells and T cells, IL-10-GFP, and PKO-IL-10-GFP were challenged with 3,000 or 5,000 PFU MCMV intraperitoneally. To study the role of IL-10 in immunocompetent mice, *Il10*^*fl*/*fl*^ and *NKp46-Cre-Il10*^*fl*/*fl*^ mice were challenged with 12,000 or 50,000 PFU MCMV. To investigate the role of IL-10 in immunocompromised mice, *PKO-Il10*^*fl*/*fl*^ and *PKO-NKp46-Cre-Il10*^*fl*/*fl*^ mice were challenged with 3,000 or 5,000 PFU MCMV. For measuring the viral titers, organs from infected mice were homogenized by MagNA Lyser (Roche Applied Science) and the lysates were diluted and overlaid on mouse embryonic fibroblasts cells for 1 h at 37°C in 2% DMEM (DMEM medium supplemented with 2% FBS, 1× penicillin/streptomycin, 2 mM L-glutamine, 10 mmol HEPES, and 50 μmol 2-mercaptoethanol). After 1 h incubation, the virus was removed from the monolayers by aspiration. The monolayers were overlaid with 1 part of DMEM containing 2% low melting agar mixed with 3 parts of 13.5% DMEM (DMEM medium supplemented with 13.5% FBS, 1× penicillin/streptomycin, 2 mM L-glutamine, 10 mmol HEPES, and 50 μmol 2-mercaptoethanol). Three days later, the cells were fixed with 10% formalin for 10 min and stained with 1% Crystal Violet for 10 min. Plaques were counted and represented as log PFU/g of organs.

### ALT and Cytokine Quantification

The presence of alanine aminotransferase (ALT) in the serum samples was determined using the Alanine Aminotransferase (ALT/GPT) Colorimetric Assay Kit (Elabscience, cat #: E-BC-K325). Samples were prepared according to the manufacturer's instructions, acquired on FilterMax F5 Multi-Mode Microplate Reader (Molecular Devices) and analyzed using the SoftMax Pro software (Molecular Devices). The production of cytokines IL-10, IFNγ, and TNFα in blood plasma or conditioned media was measured using Cytometric Bead Array (CBA) kit (BD Biosciences). Samples were prepared according to the manufacturer's instructions, acquired on LSRFortessa (BD Biosciences) and analyzed using the FCAP Array software (BD Biosciences).

### Cell Isolation, Serum, and Conditioned Media (CM)

Approximately 40 μL of peripheral blood was collected from the tail vein and washed with 1 mL PBS. The erythrocytes were lysed using 1 mL of Red Blood Cell Lysis Buffer (Roche) for about 30 s until the solution became clear. The cells were then washed with 9 mL of RPMI and filtered through a 70 μm nylon mesh. Spleens were harvested, weighed, transferred onto a 70 μm nylon cell strainer, and ground. A single-cell suspension of leukocytes was obtained following red blood cell lysis and filtration through a 70 μm nylon mesh. For the isolation of hepatic lymphocytes, livers were harvested, weighed, and transferred onto the surface of 70 μm nylon cell strainer and ground. The cells were washed three times with RPMI-1640 medium and suspended in 4 ml of 40% Percoll (GE Healthcare) and carefully overlaid onto 2 ml of 70% Percoll at room temperature (RT). The liver samples were then centrifuged at 2,400 rpm for 25 min at RT with no breaks. The top fat layer was suctioned off using a Pasteur pipette, and the lymphocyte ring was collected and resuspended in RPMI-1640 medium. The isolation of lymphocytes from the salivary glands was performed as previously described (44). The number of viable cells was determined by trypan blue exclusion. Serum was obtained following centrifugation of whole blood collected in heparin tubes and stored at −20°C until further use for cytokine analysis. To prepare CM, purified NK cells were seeded in triplicates in a flat-bottom 96-well plate at 5 × 10^4^ cells/well in RP-10 medium (RPMI-1640 medium supplemented with 10% FBS, 1× penicillin/streptomycin, 2 mM L-glutamine, 10 mmol HEPES, 50 μmol 2-mercaptoethanol) and stimulated with recombinant human (rh)IL-2 (1,000 U/mL, NCI Preclinical Repository) and recombinant mouse IL-12 (50 ng/mL, eBioscience). After 18 h of incubation at 37°C, cell-free supernatants were collected and stored at −20°C until used for cytokine analyses.

### Antibodies and Flow Cytometry

Single-cell suspensions (1 × 10^6^ cells) was incubated at 4**°**C for 15 min with α-CD16/32 (clone 2.4G2, from Bioexpress) to reduce non-specific binding. Cells were labeled with various combinations of directly conjugated monoclonal antibodies and incubated at 4°C for 25 min. The following monoclonal antibodies were used: α-NK1.1 (PK136), α-CD3 (145-2C11), α-TCRβ (H57-597), α-CD8a (53–6.7), α-CD49b (DX5), α-CD27 (LG.7F9), α-CD11b (M1/70), α-Ly49D (4E5), α-Ly49G2 (4D11), α-Ly49H (3D10), α-NKG2A-B6 (16a11), α-NKG2D (CX5), α-IL-10 (JES5-16E3), α-IFNγ (XMG1.2), and α-CD107a (1D4B) from eBioscience, α-CD19 (1D3), α-Ly49C/I (5E6), α-CD4 (RM4-5), and α-CD49a (Ha31/8) from BD Biosciences, Fixable Yellow Live/Dead from Invitrogen. For intracellular IL-10 and IFNγ measurements *in vivo*, leukocytes were harvested and incubated in RP-10 medium (RPMI-1640, 10% FBS, 1X penicillin/streptomycin, 1% L-Glutamine, 10 mM HEPES, 50 μM β-mercaptoethanol) containing 5 μg/ml brefeldin A for 4 h, followed by staining for intracellular IL-10 or IFNγ using BD Cytofix/Cytoperm protocol. The stained cells were acquired using LSRFortessa or FACSCelesta (BD Biosciences) and analyzed using Kaluza software v3 (Beckman Coulter) or FlowJo (Tree Star).

### NK Cell Enrichment and Cell Sorting

NK cells were enriched from total splenocytes by negative magnetic separation using NK cell isolation kit II (MACS Miltenyi Biotec) according to the manufacturer's instructions. Total splenocytes were labeled with α-NK1.1, α-TCRβ, and α-CD19 (to obtain T cells and B cells), while total hepatic leukocytes (to obtain NKT cells) and enriched NK cells were labeled with α-NK1.1 and α-TCRβ. The labeled cells were then flow sorted by using MoFlo XDP-sorter from Beckman Coulter (Stem Core laboratories, OHRI, Ottawa) to obtain T cells, B cells, NK cells, and NKT cells. The purified NK cells were stimulated *ex vivo* with rhIL-2 (1,000 U/ml) and IL-12 (50 ng/ml) in RP-10 medium for 18 h at 37°C and the conditioned media was harvested to measure IL-10 secretion. The purity of the fraction was >95%.

### Histology

The colon samples were harvested from 6 months old mice. The samples were flushed with PBS by using syringe and needle and immersed in 4% paraformaldehyde (PFA) for fixing with slight shaking. Fixed samples were sent to the Department of Pathology and Laboratory Medicine at the University of Ottawa where the tissues were embedded and hematoxylin and eosin (H&E) stained. Slide pictures were taken at magnifications of 10 and 20.

### Statistical Analysis

Statistical significance between two groups was determined by two-tailed unpaired student *t*-tests (^*^p < 0.05; ^**^p < 0.01; ^***^p < 0.001) using Prism Version 5 (Graph Pad Software). Statistical significance between more than two groups was determined by ANOVA. If the ANOVA rejected the null hypothesis, multiple comparisons were performed between two selected groups by a two-tailed unpaired *t*-test.

## Results

### NK Cells Are a Major Source of IL-10 During the Early Stages of MCMV Infection in B6 Immunocompetent Mouse

**Figure 1:**
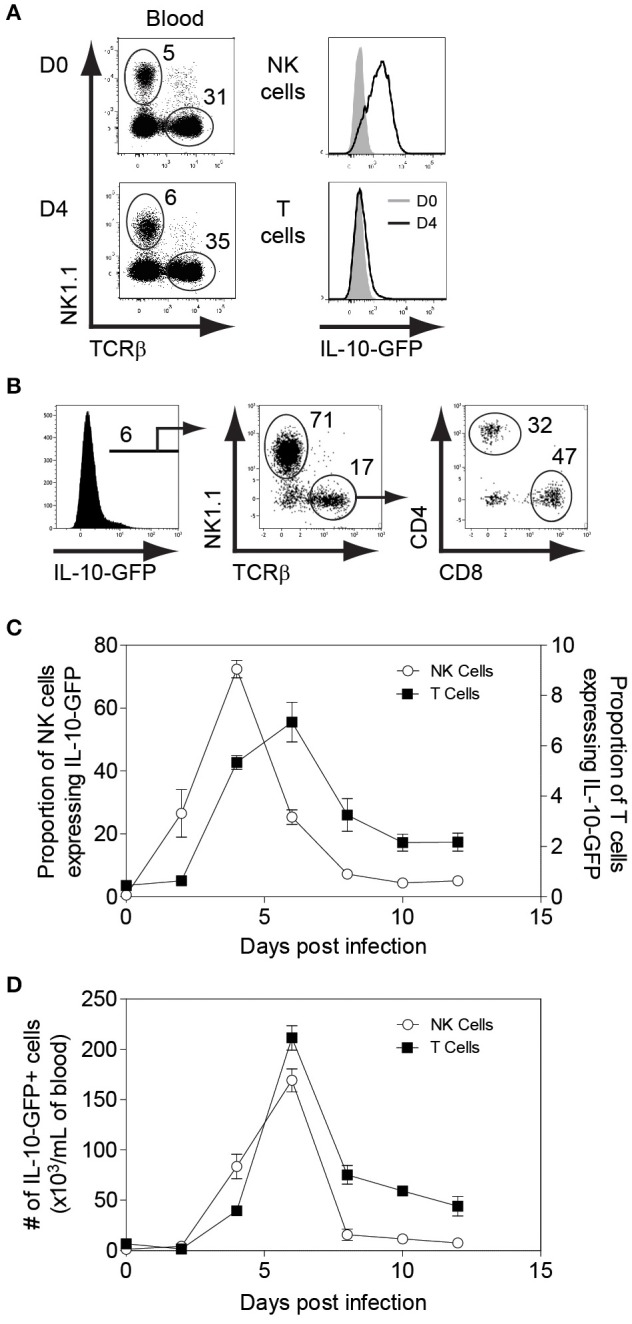
NK cells are major producers of IL-10 during the initial stages of MCMV infection. IL-10-GFP mice were either uninfected or challenged with 3,000 PFU MCMV i.p. and the peripheral blood leukocytes were analyzed on the indicated days. **(A)** The overlay plots depict IL-10-GFP expression in NK cells and T cells in the peripheral blood of uninfected (D0) and infected (D4) mice. **(B)** Back gating analysis representing IL-10 producing NK cells and T cells among total IL-10-GFP expressing cells. **(C,D)** The graphs represent the complete kinetics of IL-10-GFP expression throughout the course of MCMV infection. The proportion and number of IL-10-GFP expressing NK cells and T cells are obtained from total peripheral blood leucocytes (*n* = 3; two experiments). Data represent mean + SD.

To determine whether NK cells play an immunoregulatory role in immunocompetent mice by producing IL-10, we infected IL-10-GFP reporter mice (C57BL/6 background), which allows us to study the complete kinetics of IL-10 production during MCMV infection. Flow cytometric analysis showed that NK cells were predominantly expressing IL-10-GFP on D4 post MCMV infection in the peripheral blood (Figure 1A), whereas almost negligible IL-10-GFP expression was observed in T cells at that time. A similar trend was observed in the spleen and liver (Figures S1A,B). Recently, liver-resident NK cells defined as CD49a+CD49b– have been shown to serve as sentinels in response to infection (45). The proportions of liver-resident NK cells were dramatically reduced upon MCMV infection, and the IL-10-GFP expression was lower than that of conventional CD49a–CD49b+ NK cells (Figure S1C). Back gating analysis further confirms that NK cells in the peripheral blood are major producer of IL-10 on D4 post infection. Among total IL-10-GFP expressing cells, 71% were NK cells, and only 17% were T cells (Figure 1B). The T cells expressing IL-10-GFP were comprised of both CD4 and CD8 T cells.

The complete kinetics of peripheral blood leukocytes by flow cytometry revealed that IL-10-GFP expression by NK cells starts as early as D2 with a peak observed on D4 post-infection (Figures 1C,D). After D4, the IL-10-GFP expression slowly starts to diminish in NK cells, and returns to basal levels by D10 of infection. Although GFP expression by T cells overlapped with NK cells, only a small proportion of T cells expressed IL-10 as compared to NK cells during the initial stages of infection. The IL-10-GFP expression by T cells reached a peak on D6 before starting to decrease (Figures 1C,D). Approximately 7% of T cells were IL-10-GFP positive at D6 post-infection. Taken together, NK cells are the major producers of the regulatory cytokine IL-10 during the early stages of MCMV infection in immunocompetent mice. Furthermore, NK cells and T cells participate in a division of labor for maintaining constant levels of IL-10 during MCMV infection.

### Generation and Characterization of NK Cell-Specific *Il10*-Deficient (*NKp46-Cre-Il10^*fl*/*fl*^*) Mice

The use of neutralizing antibodies against IL-10 or its receptor has been a commonly used approach to study its function. Since various cell populations can produce IL-10 during infection and different cell subsets possess IL-10 receptor, it is difficult to characterize the importance of cell-specific IL-10 production by using neutralizing antibodies. Complete IL-10-deficient (*Il10*^−/−^) mice have also been used in previous studies to elucidate the role of IL-10 (46, 47), but the severe spontaneous inflammation observed in this mouse model limited its use for infection studies. To circumvent these disadvantages, we generated a mouse model that lacked *Il10* gene specifically in NK cells (*NKp46-Cre-Il10*^*fl*/*fl*^) by crossing an *NKp46*^*iCre*^ knock-in mouse with an *Il10* floxed mouse (*Il10*^*fl*/*fl*^).

**Figure 2:**
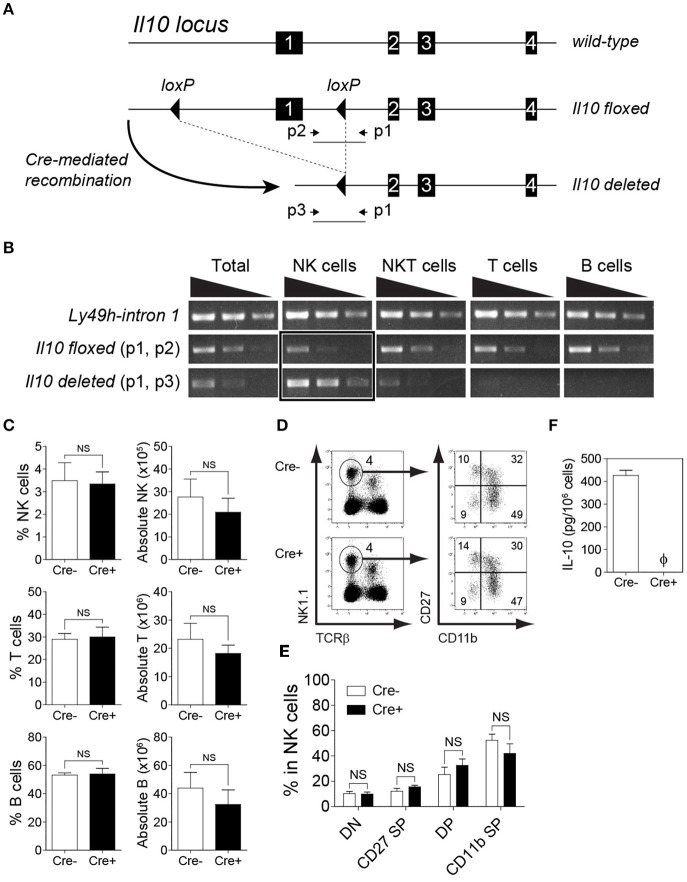
Deletion of *Il10* gene (exon 1) occurs predominantly in NK cells. Genomic deletion of *Il10* and protein expression of IL-10 in *NKp46-Cre-Il10*^*fl*/*fl*^ mice and control littermates. **(A)** In *Il10*^*fl*/*fl*^ mice, exon 1 of *Il10* gene is flanked with *loxP* sites. Crossing *Il10*^*fl*/*fl*^ mice with *NKp46-Cre* mice allows for Cre-mediated recombination to occur, leading to exon 1 deletion. The schematic diagram shows the location of gene-specific primers used to determine Cre-mediated recombination in *NKp46-Cre-Il10*^*fl*/*fl*^ mice. **(B)** Sorted NK, NKT, T, and B cells from *NKp46-Cre-Il10*^*fl*/*fl*^ mice were genotyped to determine the presence of *Il10*-floxed allele and *Il10*-deleted allele. Semi-quantitative PCR was performed on 5-fold serial dilutions of genomic DNA (50, 10, and 2 ng) from the indicated cell populations (two experiments). **(C)** Proportions and absolute numbers of NK cells, T cells, and B cells in the spleens of *Il10*^*fl*/*fl*^ (Cre−) and *NKp46-Cre-Il10*^*fl*/*fl*^ (Cre+) mice (three experiments; *n* = 7 for NK cells and T cells, *n* = 5 for B cells). **(D)** Representative plots of CD27^−^CD11b^−^ (DN), CD27^+^CD11b^−^ (CD27 SP), CD27^+^CD11b^+^ (DP) and CD27^−^CD11b^+^ (CD11b SP) populations among NK cells. **(E)** Proportions of DN, CD27 SP, DP and CD11b SP populations in *Il10*^*fl*/*fl*^ and *NKp46-Cre-Il10*^*fl*/*fl*^ mice (*n* = 3; two experiments). **(F)** Highly purified NK cells from *Il10*^*fl*/*fl*^ (Cre−) and *NKp46-Cre-Il10*^*fl*/*fl*^ (Cre+) mice were stimulated with IL-2/IL-12 for 18 h. The IL-10 production in conditioned media was analyzed by CBA (*n* = 3; two experiments). Data represent mean + SD. ns, non-significant; ϕ, not detected.

To determine whether Cre-mediated recombination occurs specifically in NK cells, we flow-sorted T cells, B cells, and NK cells from spleens and NKT cells from livers of *NKp46-Cre-Il10*^*fl*/*fl*^ mice and extracted the genomic DNA to analyze the Cre-mediated recombination by PCR. The location of the primer sets used to identify the *Il10* floxed, and *Il10* deleted alleles are shown in Figure 2A. Notably, the *Il10* deleted allele was mainly observed in NK cells (Figure 2B). Furthermore, *Il10* floxed allele was weakly amplified from NK cells but present in all other lymphocyte populations indicating that Cre mediated recombination had occurred exclusively in NK cells but not in other cell populations such as T cells, B cells and NKT cells (Figure 2B). Due to the presence of NK cells among total cells, a faint band of IL-10 deleted allele was observed in whole cells but none in T cells, B cells and only slightly in NKT cells. The *NKp46-Cre-Il10*^*fl*/*fl*^ mice were obtained at Mendelian frequency, were fertile and developed normally. Taken together, the PCR analysis confirmed that Cre-mediated recombination resulted in *Il10* gene deletion specifically in NK cells, whereas it was intact in all other lymphocyte populations.

The deletion of IL-10 in NK cells did not affect the proportion and number of immune cells at the steady-state, as the frequencies of lymphoid and myeloid subsets were comparable in the spleens of *Il10*^*fl*/*fl*^ and *NKp46-Cre-Il10*^*fl*/*fl*^ mice (Figure 2C and data not shown). In addition, the maturation of NK cells was normal in *NKp46-Cre-Il10*^*fl*/*fl*^ mice (Figures 2D,E). The expression of activating and inhibitory receptors was also comparable between the two groups of mice (Figure S2). It has been previously reported that the synergistic action of IL-2/IL-12 induces IL-10 production by NK cells (12, 48). Therefore, to confirm the loss of IL-10 protein in naïve NK cells from *NKp46-Cre-Il10*^*fl*/*fl*^ mice following *ex vivo* IL-2/IL-12 stimulation, we obtained NK cells with a purity of >95% by enrichment followed by cell sorting (Figure S3). The highly pure NK cells were subjected to *ex vivo* stimulation in the presence of IL-2/IL-12. Indeed, we could not detect any secreted IL-10 in the conditioned media of stimulated NK cells from *NKp46-Cre-Il10*^*fl*/*fl*^ mice (Figure 2F). This further confirmed the loss of IL-10 production by NK cells in *NKp46-Cre-Il10*^*fl*/*fl*^ mice, whereas IL-10 production was intact in control *Il10*^*fl*/*fl*^ mice.

### *NKp46-Cre-Il10^*fl*/*fl*^* Mouse Does Not Show Signs of Spontaneous Inflammation

**Figure 3:**
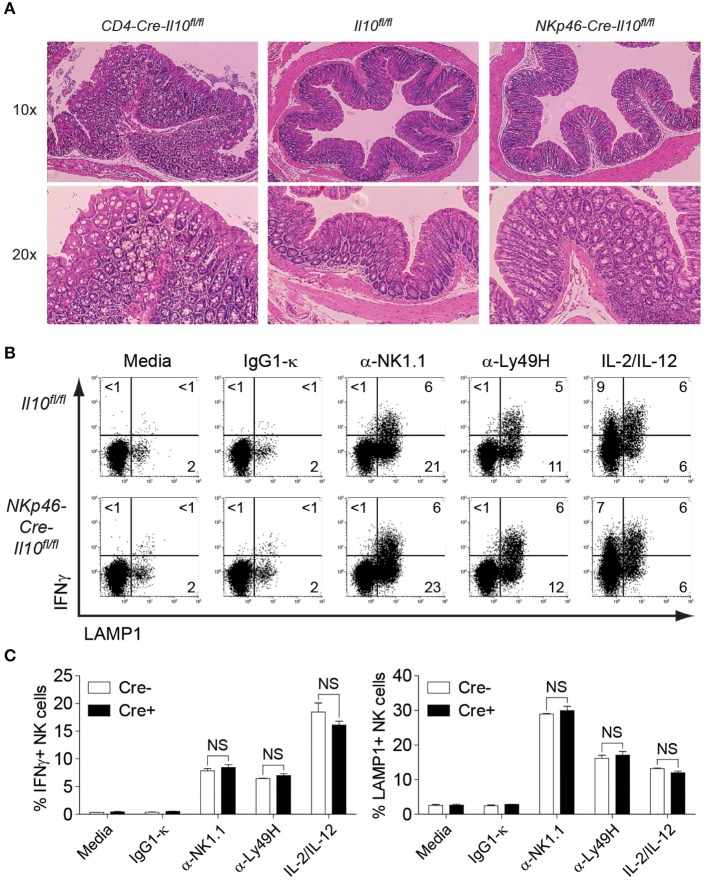
No signs of spontaneous inflammation observed in *NKp46-Cre-Il10*^*fl*/*fl*^ mice. **(A)** Colon histology of 6 months old *CD4-Cre-Il10*^*fl*/*fl*^, *Il10*^*fl*/*fl*^, and *NKp46-Cre-Il10*^*fl*/*fl*^ mice is performed (*n* = 3; two experiments). Tissues are fixed in 10% formaldehyde and paraffin-embedded. Histological sections are shown at the indicated magnification. **(B)** Representative plots of intracellular IFNγ and LAMP1 expression on NK cells from *Il10*^*fl*/*fl*^ and *NKp46-Cre-Il10*^*fl*/*fl*^ mice upon stimulation with the indicated antibodies or cytokines. **(C)** Percentage of IFNγ-producing and LAMP1-expressing NK cells from *Il10*^*fl*/*fl*^ and *NKp46-Cre-Il10*^*fl*/*fl*^ mice upon stimulation with the indicated antibodies or cytokines (*n* = 2; two experiments). Data represent mean + SD. ns, non-significant.

*Il10*^−/−^ mice develop chronic enterocolitis characterized by excessive hyperplasia of the mucosal epithelium (47). Previous studies with *CD4-Cre-Il10*^*fl*/*fl*^ mice showed the spontaneous onset of inflammatory bowel disease (IBD) as mice aged, suggesting the crucial role of T cell-derived IL-10 in protecting mice from excessive inflammation. The most prominent histological changes have been observed in the colon of *CD4-Cre-Il10*^*fl*/*fl*^ mice (41). Although Tregs have been known to play a central role in suppressing immune inflammation, it is possible that additional IL-10 sources are required to restrain spontaneous inflammation. As NK cells from *NKp46-Cre-Il10*^*fl*/*fl*^ mouse do not produce IL-10, lack of this early regulatory function may render this mouse highly susceptible to spontaneous inflammation. Since the colon is the most affected part during gut inflammation as exemplified in earlier studies, we examined the colon histology in *NKp46-Cre-Il10*^*fl*/*fl*^ mice at 6 months of age and compared it with mice lacking IL-10 in T cells (*CD4-Cre-Il10*^*fl*/*fl*^). H&E staining of colon from control and *NKp46-Cre-Il10*^*fl*/*fl*^ mice showed normal colon anatomy characterized by highly organized crypt structure and surface epithelium with no signs of spontaneous inflammation in *NKp46-Cre-Il10*^*fl*/*fl*^ mice as opposed to *CD4-Cre-Il10*^*fl*/*fl*^ mice that exhibited massive hyperplasia and disorganized crypt structure indicative of spontaneous inflammation as previously reported (41) (Figure 3A).

To evaluate the activation status of NK cells in young mice, enriched NK cells from 8 weeks old naïve control and *NKp46-Cre-Il10*^*fl*/*fl*^ mice were stimulated by various surface-bound stimulations such as α-NK1.1, α-Ly49H, and IL-2/IL-12 and their effector functions were analyzed. NK cells from control and *NKp46-Cre-Il10*^*fl*/*fl*^ mice showed comparable levels of IFNγ production and LAMP-1 (a marker of degranulation) upon various stimulations (Figures 3B,C), suggesting that NK cells from control and *NKp46-Cre-Il10*^*fl*/*fl*^ mice are phenotypically and functionally similar in the naïve state. Taken together, we have successfully generated a NK cell-specific *Il10*-deficient mouse that is free of spontaneous inflammation and hence is an ideal tool to elucidate the immunoregulatory role of NK cell-derived IL-10 during microbial infection.

### *NKp46-Cre-Il10^*fl*/*fl*^* Mouse Shows Comparable Viral Clearance and T Cell Response During Acute MCMV Infection of Immunocompetent Mice

**Figure 4:**
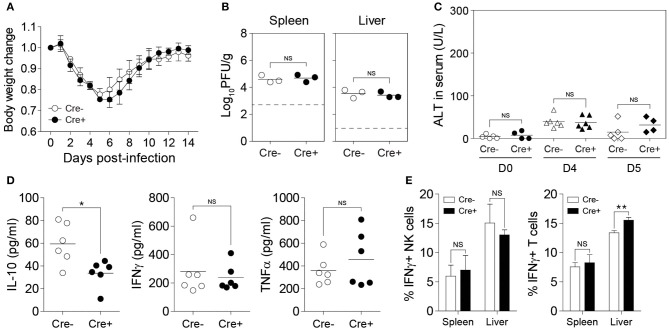
NK cell-derived IL-10 is dispensable during acute MCMV infection. *Il10*^*fl*/*fl*^ (Cre−) and *NKp46-Cre-Il10*^*fl*/*fl*^ (Cre+) mice were either uninfected or given 12,000 PFU or 50,000 PFU MCMV i.p. for the following analyses: **(A)** Body weight change of infected mice relative to their initial body weight at day 0 post-infection (*n* = 3; two experiments). **(B)** Viral titer in the spleen of *Il10*^*fl*/*fl*^ and *NKp46-Cre-Il10*^*fl*/*fl*^ mice at day 5 post-infection (*n* = 3; two experiments). **(C)** Serum levels of ALT enzyme at the indicated days post-infection (three experiments; *n* = 5 for D0 Cre−, *n* = 4 for D0 Cre+, *n* = 6 for D4 Cre−, *n* = 6 for D4 Cre+, *n* = 5 for D5 Cre−, *n* = 4 for D5 Cre+). **(D)** Serum levels of IL-10, IFNγ, and TNFα were measured at day 4 post-infection (*n* = 6; two experiments). **(E)** NK cells and T cells from the spleens and livers of *Il10*^*fl*/*fl*^ and *NKp46-Cre-Il10*^*fl*/*fl*^ mice at day 5 post-infection were stained for IFNγ production (*n* = 3; two experiments). Data represent mean + SD. ns, non-significant; *p < 0.05; **p < 0.01.

The ability of NK cells to produce IL-10 during MCMV infection was demonstrated above (Figure 1), however, whether NK cell-derived IL-10 can contribute in modulating the adaptive immune response and dictating the outcome of disease has never been clearly demonstrated. Having successfully generated an NK cell-specific *Il10*-deficient mouse model, we decided to investigate the role of NK cell-derived IL-10 during acute viral infection. We infected *Il10*^*fl*/*fl*^ and *NKp46-Cre-Il10*^*fl*/*fl*^ mice with MCMV, and monitored body weight changes daily. By day 5 post-infection, the mice had lost as much as 25% of their initial body weight, before starting to gain back their weight. These mice were able to recover their initial weight by day 12 post-infection. NK cell-derived IL-10 did not seem to play a critical role in preventing the weight loss, as *NKp46-Cre-Il10*^*fl*/*fl*^ mice and their control littermates *Il10*^*fl*/*fl*^ displayed similar body weight change (Figure 4A). Notably, the presence of NK cell-derived IL-10 did not improve viral clearance, as *NKp46-Cre-Il10*^*fl*/*fl*^ mice and their control littermates *Il10*^*fl*/*fl*^ had a similar viral burden in their spleens at D5 post-infection (Figure 4B). Moreover, *NKp46-Cre-Il10*^*fl*/*fl*^ mice and their control littermates *Il10*^*fl*/*fl*^ showed similar levels of the liver enzyme alanine aminotransferase (ALT) in the serum (Figure 4C), suggesting that NK cell-derived IL-10 does not regulate liver damage in immunocompetent mice.

To determine whether NK cell-specific IL-10 ablation can lead to a reduction in the systemic levels of IL-10 during viral infection, we measured the IL-10 levels in the peripheral blood of MCMV-infected mice. Interestingly, although we were able to successfully delete IL-10 in NK cells using *NKp46-Cre-Il10*^*fl*/*fl*^ mice as shown in Figure 2, the IL-10 levels in the serum were only slightly decreased in *NKp46-Cre-Il10*^*fl*/*fl*^ mice at day 4 post-infection (Figure 4D). In addition, the serum levels of IFNγ and TNFα were similar between the two groups. In order to determine whether NK cell-derived IL-10 can regulate T cell activation during infection, we measured the IFNγ production by T cells. However, in the absence of NK cell-derived IL-10, the production of IFNγ by T cells in the spleens of infected mice was not altered and only slightly increased in the liver (Figure 4E). Taken together, these results suggest that the IL-10 produced by NK cells in immunocompetent mice during an acute MCMV infection is dispensable for viral clearance and is not critical in regulating the T cell response.

The salivary gland environment is highly immunosuppressive, making it an immune-privileged site for viral persistence (49). Both MCMV and HCMV are known for establishing a lifelong latent infection inside the salivary glands and therefore act as a natural source of horizontal viral transmission (24). Several studies have reported a role for IL-10 in maintaining the immunosuppressive environment inside the salivary glands (19, 35). To study the effect of NK cell-derived IL-10 in maintaining an immunosuppressive environment and viral persistence inside the salivary glands, we infected *Il10*^*fl*/*fl*^ and *NKp46-Cre-Il10*^*fl*/*fl*^ mice and analyzed the T cell response in salivary glands on day 16 post-infection. Notably, we did not observed any difference in the viral burden or T cell response between the groups (Figures S4A,B). In fact, using MCMV-infected IL-10-GFP mice, we observed that NK cells are poor producers of IL-10 in the salivary gland whereas CD4 T cells are major producers of IL-10 (Figure S4C).

### Perforin-Deficient Mice Have More Sustained Production of IL-10

**Figure 5:**
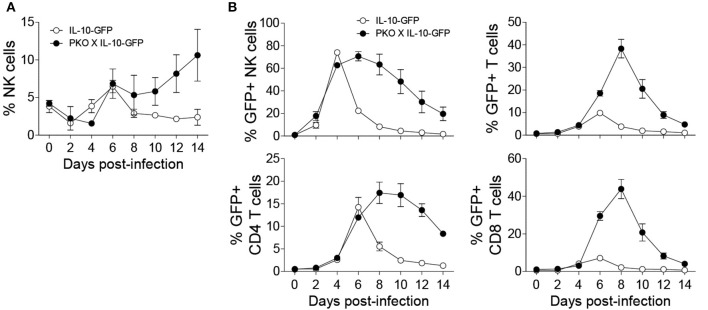
NK cells from *perforin*-deficient (PKO) mice express more sustained levels of IL-10 during MCMV infection. IL-10-GFP and PKO-IL-10-GFP mice were either uninfected or given 5,000 PFU MCMV i.p. and the peripheral blood leukocytes were analyzed on the indicated days. **(A)** The proportion of NK cells in the blood of IL-10-GFP and PKO-IL-10-GFP infected mice throughout the course of MCMV infection. **(B)** The graphs represent the complete kinetics of IL-10-GFP expression in NK cells and T cells from the peripheral blood of IL-10-GFP (*n* = 4) and PKO-IL-10-GFP (*n* = 4) infected mice at the indicated days post-infection (two experiments). Data represent mean + SD.

Since the need for regulatory pathways is well-recognized during persistent infections in which immune cells are often highly activated, we decided to examine the importance of NK cell-specific IL-10 during a persistent MCMV model. To analyze the kinetics of IL-10 production by NK cells in perforin-deficient mice, we crossed perforin-deficient (PKO) mice with IL-10-GFP mice (PKO X IL-10-GFP). NK cells from perforin-deficient mice undergo extensive proliferation during MCMV infection, as compared to immunocompetent mice (Figure 5A). Notably, NK cells from perforin-deficient mice produced more sustained levels of IL-10 (Figure 5B). IL-10 production by NK cells in IL-10-GFP mice peaked at day 4 post-infection before declining rapidly by day 6 post-infection. However, NK cells in PKO X IL-10-GFP mice produced high amounts of IL-10 from day 4 to day 8 post-infection before starting to decrease. Similarly, T cells from PKO mice also produced more sustained levels of IL-10. In IL-10-GFP mice, IL-10 production by T cells was acute and peaked at day 6 post-infection. On the other hand, in PKO mice, IL-10 production by T cells was higher and peaked at day 8 post-infection. In addition, CD4 T cells showed a more sustained production of IL-10, while CD8 T cells showed an acute production of IL-10. Taken together, NK and T cells from PKO mice produce more sustained levels of IL-10 during MCMV infection, suggesting a critical role of IL-10 during persistent infection.

### NK Cell-Derived IL-10 in Perforin-Deficient Mice Prevents Liver Damage During Sustained MCMV Infection

**Figure 6:**
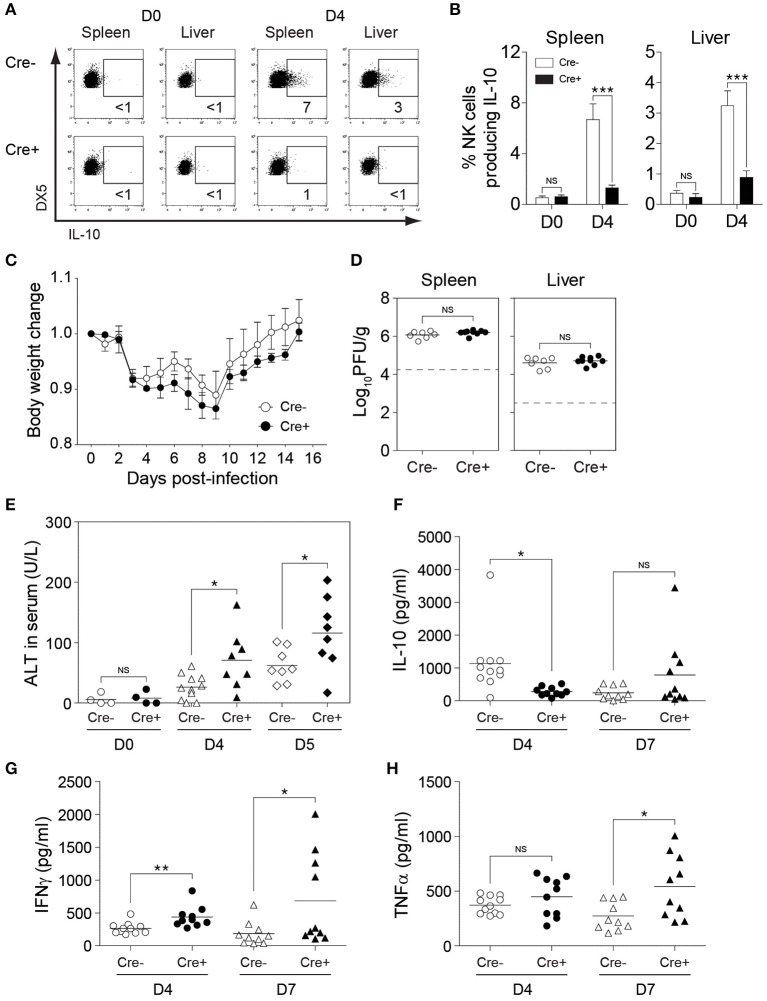
NK cell-derived IL-10 provides protection against liver damage during sustained MCMV infection. *PKO-Il10*^*fl*/*fl*^ (Cre−) and *PKO-NKp46-Cre-Il10*^*fl*/*fl*^ (Cre+) mice were either uninfected or given 3,000 PFU or 5,000 PFU MCMV i.p. for the following analyses: **(A,B)** Proportion of NK cells (DX5+TCRβ-) expressing IL-10 in uninfected and infected mice at day 4 post-infection (two experiments; *n* = 4 for D0 Cre−, *n* = 4 for D0 Cre+, *n* = 7 for D4 Cre−, *n* = 5 for D4 Cre+). **(C)** Body weight change of *PKO-Il10*^*fl*/*fl*^ (*n* = 4) and *PKO-NKp46-Cre-Il10*^*fl*/*fl*^ (*n* = 3) infected mice relative to their initial body weight at day 0 post-infection (three experiments). **(D)** Viral titer in the spleens and livers of *PKO-Il10*^*fl*/*fl*^ (*n* = 7) and *PKO-NKp46-Cre-Il10*^*fl*/*fl*^ (*n* = 9) mice at day 5 post-infection (two experiments). **(E)** Serum levels of ALT enzyme at the indicated days post-infection (four experiments; *n* = 4 for D0 Cre−, *n* = 4 for D0 Cre+, *n* = 11 for D4 Cre−, *n* = 8 for D4 Cre+, *n* = 8 for D5 Cre−, *n* = 8 for D5 Cre+). **(F–H)** Serum levels of IL-10, IFNγ, and TNFα were measured at day 4 and day 7 post-infection (three experiments; *n* = 11 for D4 Cre−, *n* = 10 for D4 Cre+, *n* = 10 for D7 Cre−, *n* = 10 for D7 Cre+). Data represent mean + SD. ns, non-significant; *p < 0.05; **p < 0.01; ***p < 0.001.

To investigate the role of NK cell-derived IL-10 in perforin-deficient mice during MCMV infection, we generated *Il10*^*fl*/*fl*^ and *NKp46-Cre-Il10*^*fl*/*fl*^ mice under a perforin-deficient background to give rise to *PKO-NKp46-Cre-Il10*^*fl*/*fl*^ mice and their control littermates *PKO-Il10*^*fl*/*fl*^ which lack the Cre recombinase. These mice were infected with MCMV, and their body weight was monitored daily. NK cells from *PKO-NKp46-Cre-Il10*^*fl*/*fl*^ mice failed to produce IL-10 during MCMV infection, as measured by intracellular staining of IL-10 (Figures 6A,B). PKO mice challenged with MCMV suffered slightly increased body weight loss when NK cells were unable to produce IL-10 (Figure 6C). Notably, the inability of NK cell to produce IL-10 did not influence the viral burden in MCMV-infected PKO mice, as *PKO-NKp46-Cre-Il10*^*fl*/*fl*^ had similar virus titer in their spleens and livers as compared with their control littermates *PKO-Il10*^*fl*/*fl*^ at D5 post-infection (Figure 6D). Despite that, the absence of NK cell-derived IL-10 led to increased liver damage, based on the levels of the liver enzyme ALT in the serum of infected mice (Figure 6E), suggesting that NK cell-produced IL-10 prevents immunopathology.

**Figure 7:**
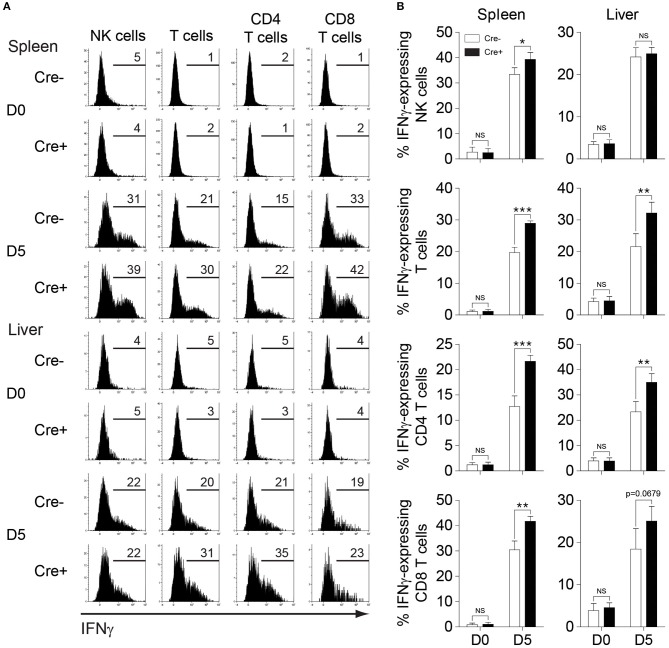
NK cell-derived IL-10 regulates T cell activation during sustained MCMV infection. *PKO-Il10*^*fl*/*fl*^ (Cre−) and *PKO-NKp46-Cre-Il10*^*fl*/*fl*^ (Cre+) mice were either uninfected or given 5,000 PFU MCMV i.p. **(A,B)** NK cells, CD4 T cells, and CD8 T cells from the spleens and livers of *PKO-Il10*^*fl*/*fl*^ (*n* = 4) and *PKO-NKp46-Cre-Il10*^*fl*/*fl*^ (*n* = 4) mice at day 5 post-infection were stained for IFNγ production (three experiments). Data represent mean + SD. ns, non-significant; *p < 0.05; **p < 0.01; ***p < 0.001.

Next, we investigated whether NK cell-specific IL-10 ablation in perforin-deficient mice leads to a reduction in the systemic levels of IL-10 during infection. Thus, we measured the IL-10 levels in the peripheral blood of MCMV-infected mice. Indeed, the IL-10 levels in the serum were reduced in *PKO-NKp46-Cre-Il10*^*fl*/*fl*^ mice at D4 post-infection, as compared to their control littermates (Figure 6F), however this difference was not observed at D7 post-infection. Notably, the levels of IFNγ and TNFα were increased in the serum of *PKO-NKp46-Cre-Il10*^*fl*/*fl*^ mice (Figures 6G,H), indicating that NK cell-derived IL-10 can regulate the systemic levels of pro-inflammatory cytokines during a sustained MCMV infection. To determine whether the IL-10 produced by NK cells can directly regulate T cell activation, we measured the proportions of IFNγ-producing CD4 and CD8 T cells in the spleens and livers of infected mice. Notably, T cells showed increased production of IFNγ in *PKO-NKp46-Cre-Il10*^*fl*/*fl*^ mice, as compared to their control littermates (Figures 7A,B and Figure S5), indicating that NK cells produce IL-10 to control the activation status of T cells during MCMV infection.

## Discussion

NK cells have been well-recognized as potent pro-inflammatory cells due to their capability of secreting cytokines such as IFNγ and TNFα that limit microbial growth during the initial stages of infections (5). Furthermore, NK cell-derived IFNγ helps to shape the T cell response in the lymph nodes, possibly through a direct interaction between naïve T cells and NK cells migrating from the site of inflammation to secondary lymphoid organs or by an indirect effect on DCs (50). Notably, several reports have demonstrated that NK cells are potent producers of IL-10, suggesting their role in regulating the immune response (36, 51). In this study, we have demonstrated that NK cells are a key source of IL-10 during the early phases of MCMV infection and that NK cell-derived IL-10 is important to protect the host against liver damage during a persistent virus infection. To evaluate IL-10 production, we employed IL-10-GFP reporter mice (C57BL/6 background). The use of this IL-10-GFP reporter mouse to measure IL-10 expression is advantageous over the intracellular staining of IL-10 because the signals obtained by intracellular staining are generally weak and challenging to interpret. Since the *Gfp* gene is inserted in the 3' untranslated end of the *Il10* gene locus in the reporter mouse, *Gfp* expression is directly correlated to *Il10* gene expression (40). Such mice have been previously used to study the role of intestinal T cell-derived IL-10 in regulating gut inflammation (40), B cell-derived IL-10 during autoimmunity (45) and IL-10 mediated regulation of liver inflammation during acute MCMV infection (28).

Previously, we have demonstrated that IL-10 blockade in persistent MCMV infection resulted in an elevated CD8 T cell response, higher levels of circulating pro-inflammatory cytokines IFNγ and TNFα, and increased viral burden, which led to poor survival of the host (36). Since different immune cell types can participate in the production of circulating IL-10, blocking IL-10 functions using neutralization antibodies against IL-10 limits our understanding of the role of IL-10 from a particular cell subset. To overcome the limitation, using the Cre induced recombination of *Il10* genomic locus, we generated *NKp46-Cre-Il10*^*fl*/*fl*^ mice to elucidate the regulatory role of NK cells during viral infection. In the *NKp46*^*iCre*^ knock-in mouse, Cre recombinase is expressed under the control of the NKp46 promoter, which is mainly found in NK cells (52–54). The faithful expression of the *Cre* gene in NK cells has been confirmed previously (42). *Il10* floxed mice carry *loxP* sites flanking exon 1 of the *Il10* gene and have been used previously to investigate the effect of T cell (41) and Treg (55) specific IL-10 ablation on the immune response. We confirmed the specific IL-10 deficiency in NK cells from *NKp46-Cre-Il10*^*fl*/*fl*^ mice at the genomic and protein level. Whether spontaneous inflammation develops in the *NKp46-Cre-Il10*^*fl*/*fl*^ mice was intriguing because both conventional *Il10*^−/−^mice and T cell-specific *Il10-*deficient mice on the C57BL/6 background develop rectal prolapse and diarrhea that are indicators of severe spontaneous intestinal inflammation at 6 months of age (47). Although Tregs have been known to play a central role in suppressing immune-mediated inflammation, it is possible that additional sources of IL-10 are required to restrain spontaneous gut inflammation. Our analysis of NK cell adhesion molecules and activation status *in vitro* showed the absence of any aberrant inflammation in *NKp46-Cre-Il10*^*fl*/*fl*^ mice. Taken together, the *NKp46-Cre-Il10*^*fl*/*fl*^ mouse is a useful tool to study the regulatory role of NK cells and can be employed in various infection studies.

To dissect the immunoregulatory role of NK cells during infection, *NKp46-Cre-Il10*^*fl*/*fl*^ and their control littermates were infected with MCMV. No significant impact of NK cell-derived IL-10 on the T cell response and the disease outcome was observed. Despite the NK cell-specific IL-10 ablation, the serum levels of IL-10 were only slightly reduced, suggesting that the production of IL-10 by other cell types such as macrophages, dendritic cells, and Tregs might compensate for the absence of NK cell-specific IL-10 and account for the similar phenotype observed. Previously, CD4 T cell-derived IL-10 was shown to be critical in establishing immunosuppression in the salivary gland, allowing for viral persistence (19, 35). Since NK cells are known to be present in the salivary gland (44), we investigated whether NK cells in the salivary gland could also play an immunosuppressive role to allow viral persistence. However, we observed comparable viral loads and IFNγ response of T cells on day 16 post-infection between *NKp46-Cre-Il10*^*fl*/*fl*^ mice and their control littermates, demonstrating that NK cell-derived IL-10 is dispensable in establishing immunosuppression in the salivary gland during MCMV infection.

Regulatory pathways on immune cells have been suggested to be more critical during persistent infections (56, 57). We have previously demonstrated that perforin-deficient mice show elevated virus burdens accompanied by the extensive proliferation of NK cells during MCMV infection (36). Interestingly, IL-10 production is positively regulated in proliferating NK cells via the epigenetic modification of the IL-10 locus (58). Hence, in NK cells that undergo proliferation, the IL-10 gene would be more accessible for transcription, allowing for increased IL-10 production. In order to investigate the importance of NK cell-specific IL-10 during a persistent MCMV model, we crossed perforin-deficient mice with IL-10-GFP reporter mice, and demonstrated that NK cells produce more sustained levels of IL-10 in mice deficient in perforin. Then, we showed that in *PKO-NKp46-Cre-Il10*^*fl*/*fl*^ mice, the absence of IL-10 by NK cells resulted in detrimental disease outcome, as exemplified by increased body weight loss, and worsened liver damage accompanied by highly activated T cells. Taken together, NK cell-derived IL-10 is important in regulating the T cell response and in protecting the host from excessive immune-mediated pathology during persistent infection.

A previous study conducted to characterize the inflammatory response in MCMV infected liver has supported the role of cytokines and chemokines in modulating immune-mediated pathology in infected organs (28). This study demonstrated elevated IFNγ production suggesting the role of pro-inflammatory cytokines in mediating pathology at the site of infection. It is conceivable that IL-10 being a potent regulatory cytokine can protect the organ architecture from the immune-mediated pathology by limiting the production of pro-inflammatory cytokines.

The immunoregulatory function of IL-10 production by numerous other cell types has been demonstrated. Previous work demonstrated that myeloid cells produce IL-10 to promote the survival of the host upon *Citrobacter rodentium* infection by limiting IL-23 production (59). In another study, B cell production of IL-10 was shown to regulate the activation of antigen-specific CD8 T cells and the expansion of plasma cells (38). Moreover, the loss of IL-10 function either by genetic deletion of *Il10* gene or by blocking IL-10 receptor results in the accumulation of IFNγ producing CD4 T cells and reduced viral burden, suggesting a role of IL-10 in maintaining an immunosuppressive environment (19, 35). In this report, our data demonstrated that NK cell-derived IL-10 is indispensable in protecting the host from liver damage during persistent infection. Therefore, inducing NK cell regulatory pathway during autoimmune diseases, transplantation-related disorders, and infections with overwhelming inflammation can help restrain excessive immune-mediated inflammatory diseases.

The NK cells are one of the predominant populations of lymphocytes found during early gestation. Interestingly, the human uterine NK cells (uNK) have also been shown to produce IL-10 via IL-2/IL-12 mediated stimulation. The current speculation about uNK derived IL-10 during pregnancy is the protection of fetus from the harmful maternal immune response by inhibiting production of deleterious pro-inflammatory cytokines (60). Therefore, investigating the immunoregulatory functions of NK cells will generate valuable insights into NK cell-mediated modulation of innate and adaptive immune responses, improve our ability to monitor their role in immune suppression and viral persistence, and perhaps, provide new methods for NK cell-based therapies.

## Data Availability Statement

All datasets generated for this study are included in the article/Supplementary Material.

## Ethics Statement

The animal study was reviewed and approved by The Animal Care Committee, University of Ottawa. Written informed consent was obtained from the owners for the participation of their animals in this study.

## Author Contributions

AA, AK, and SA performed experiments. AA and AK analyzed the data. AA, AK, and S-HL designed the study and wrote the manuscript. S-HL obtained funding.

### Conflict of Interest

The authors declare that the research was conducted in the absence of any commercial or financial relationships that could be construed as a potential conflict of interest.
