# Supplementary material for: Natural Killer Cell-Derived IL-10 Prevents Liver Damage During Sustained Murine Cytomegalovirus Infection
Source: Front Immunol. 2019 Nov 15;10:2688. doi: 10.3389/fimmu.2019.02688 (PMC6873346; doi:10.3389/fimmu.2019.02688)
Supplement: Supplementary file 1 [file Data_Sheet_1.pdf]

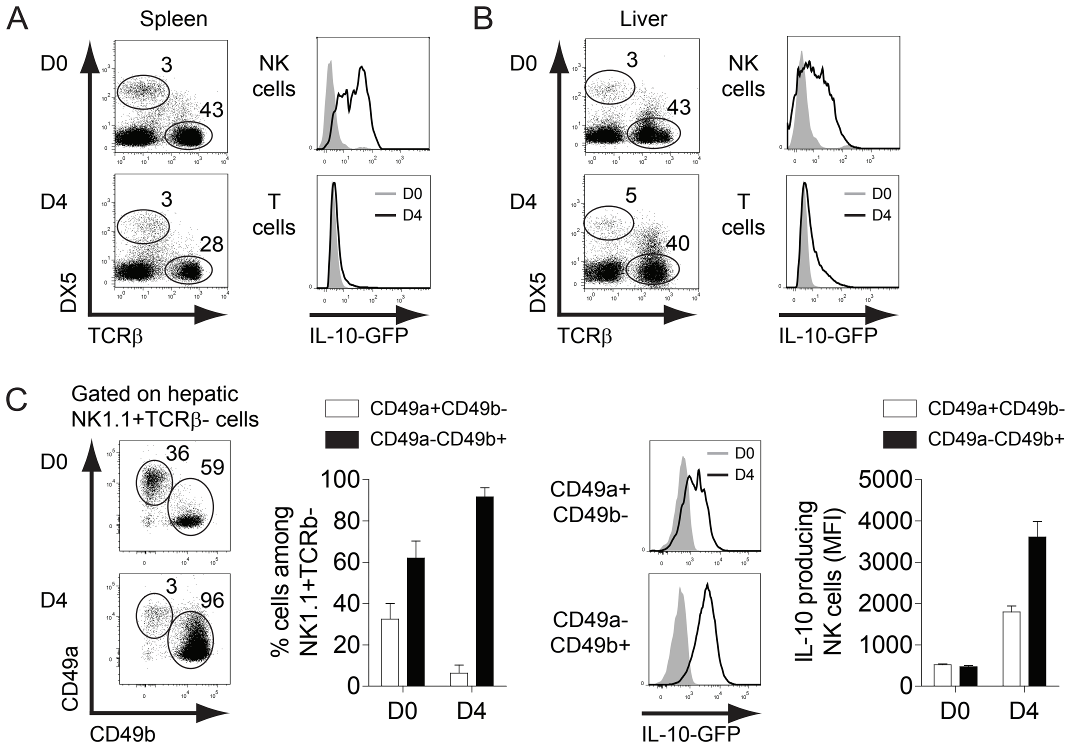

Figure S1. NK cells are major producers of IL-10 during the early stages of MCMV infection. IL-10-GFP mice were either uninfected or challenged with MCMV i.p. and the splenic and hepatic lymphocytes were analyzed on the indicated days. The overlay plots depict IL-10-GFP expression in NK cells and T cells in the spleens (A) and livers (B) of uninfected (D0) and infected (D4) mice. (C) The proportion of liver-resident NK cells (CD49a+CD49b-) and conventional NK cells (CD49a-CD49b+) among total NK cells and their production of IL-10 are shown in the livers of uninfected (D0) and infected (D4) mice ($n = 4$). Data are from one experiment representative of two independent experiments.

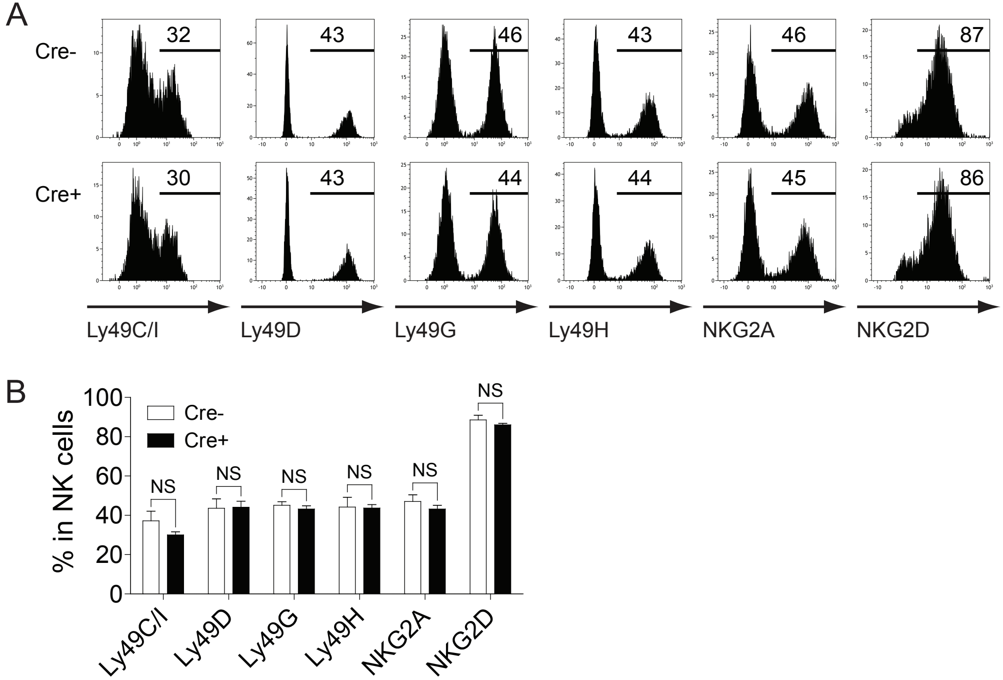

Figure S2. Phenotypic characterization of NK cells in the spleens of *Il10*$^{fl/fl}$ (Cre-) and *NKp46-Cre-Il10*$^{fl/fl}$ (Cre+) mice. (A) Representative plots of several activating and inhibitory receptors in NK cells of *Il10*$^{fl/fl}$ and *NKp46-Cre-Il10*$^{fl/fl}$ mice. (B) Proportions of NK cells expressing the indicated surface receptors. Data are from one experiment representative of two independent experiments, with three mice per group. Data represent mean + SD. ns: non significant.

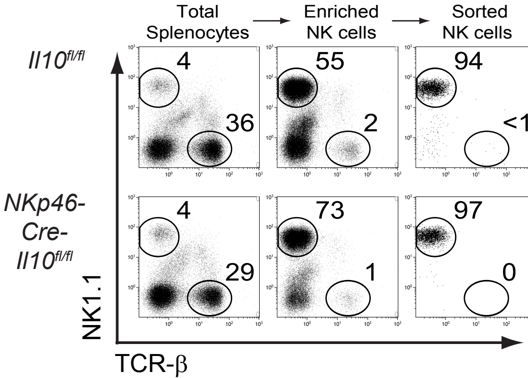

Figure S3. NK cells purity following magnetic cell enrichment and flow sorting. Flow cytometric analysis shows the purity of NK cells from *Il10$^{fl/fl}$* and *NKp46-Cre-Il10$^{fl/fl}$* mice after NK cell enrichment followed by sorting. Data are from one experiment representative of two independent experiments.

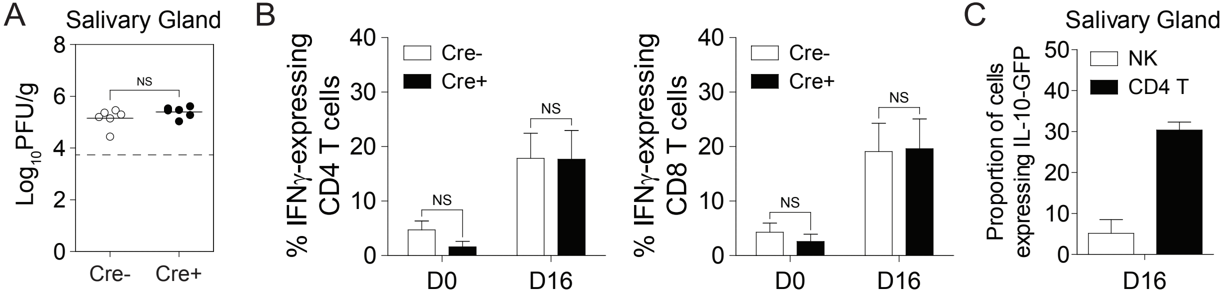

Figure S4. NK cell derived IL-10 is not important to control viral persistence in the salivary gland during MCMV infection. *Il10^fl/fl^* (Cre-) and *NKp46-Cre-Il10^fl/fl^* (Cre+) mice were either uninfected or given 50,000 PFU MCMV i.p. (A) Viral titer in the salivary glands of *Il10^fl/fl^* and *NKp46-Cre-Il10^fl/fl^* mice at day 16 post-infection. (B) CD4 T cells and CD8 T cells from the salivary glands of *Il10^fl/fl^* and *NKp46-Cre-Il10^fl/fl^* mice at day 16 post-infection were stained for IFNγ production. (C) Proportions of NK cells and CD4 T cells from the salivary glands of IL-10-GFP mice infected with 5,000 PFU MCMV i.p. at day 16 post-infection. For A, data are from one experiment representative of three independent experiments, with six mice per group. For B, data are pooled from three independent experiments, with four to thirteen mice per group. For C, data are from one experiment representative of two independent experiments, with two mice per group. Data represent mean + SD. ns: non significant.

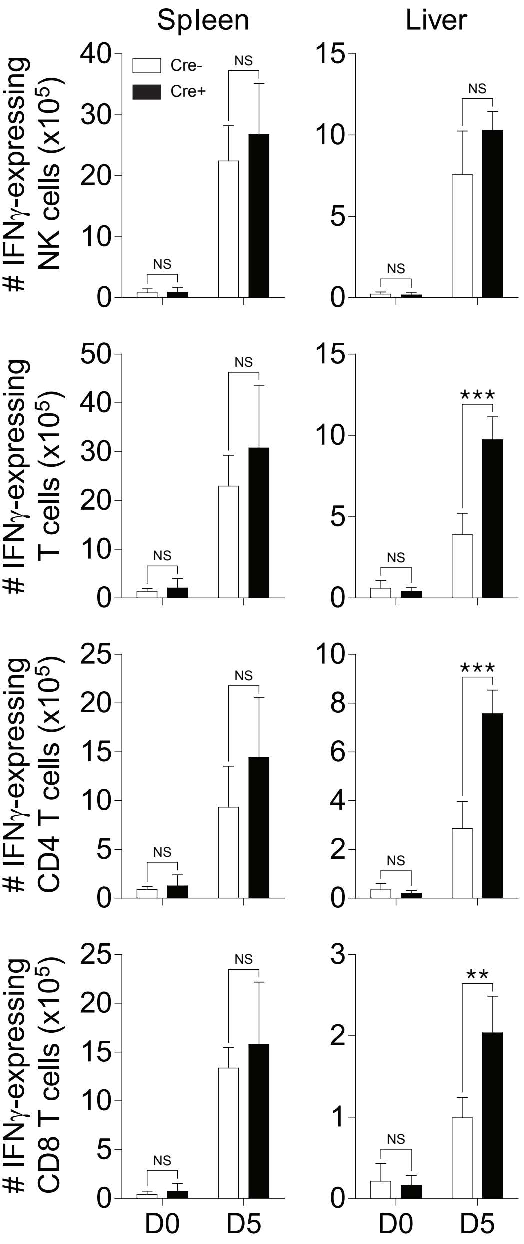

Figure S5. NK cell-derived IL-10 regulates T cell activation during sustained MCMV infection. *PKO-Il10*$^{fl/fl}$ (Cre-) and *PKO-NKp46-Cre-Il10*$^{fl/fl}$ (Cre+) mice were either uninfected or given 5,000 PFU MCMV i.p. Graphs indicate the number of NK cells, CD4 T cells, and CD8 T cells producing IFNγ in the spleens and livers of *PKO-Il10*$^{fl/fl}$ and *PKO-NKp46-Cre-Il10*$^{fl/fl}$ mice at day 5 post-infection. Data are from one experiment representative of three independent experiments, with four mice per group. Data represent mean + SD. ns: non-significant; $^{**}p < 0.01$; $^{***}p < 0.001$.
